# Strong Genetic Differentiation of Submerged Plant Populations across Mountain Ranges: Evidence from *Potamogeton pectinatus* in Iran

**DOI:** 10.1371/journal.pone.0161889

**Published:** 2016-08-25

**Authors:** Shabnam Abbasi, Saeed Afsharzadeh, Hojjatollah Saeidi, Ludwig Triest

**Affiliations:** 1 Department of Biology, Faculty of Science, University of Isfahan, 81746-73441, Isfahan, Iran; 2 Laboratory of Plant Biology and Nature Management (APNA), Department of Biology, Vrije Universiteit Brussel (VUB), Pleinlaan 2, B-1050, Brussels, Belgium; National Cheng Kung University, TAIWAN

## Abstract

Biogeographic barriers for freshwater biota can be effective at various spatial scales. At the largest spatial scale, freshwater organisms can become genetically isolated by their high mountain ranges, vast deserts, and inability to cross oceans. Isolation by distance of aquatic plants is expected to be stronger across than alongside mountain ridges whereas the heterogeneity of habitats among populations and temporary droughts may influence connectivity and hamper dispersal. Suitable aquatic plant habitats became reduced, even for the widespread submerged *Potamogeton pectinatus* L. (also named *Stuckenia pectinata*) giving structure to various aquatic habitats. We compared the level of genetic diversity in a heterogeneous series of aquatic habitats across Iran and tested their differentiation over distances and across mountain ranges (Alborz and Zagros) and desert zones (Kavir), with values obtained from temperate region populations. The diversity of aquatic ecosystems across and along large geographic barriers provided a unique ecological situation within Iran. *P*. *pectinatus* were considered from thirty-six sites across Iran at direct flight distances ranging from 20 to 1,200 km. Nine microsatellite loci revealed a very high number of alleles over all sites. A PCoA, NJT clustering and STRUCTURE analysis revealed a separate grouping of individuals of southeastern Iranian sites and was confirmed by their different nuclear ITS and cpDNA haplotypes thereby indicating an evolutionary significant unit (ESU). At the level of populations, a positive correlation between allelic differentiation *D*_est_ with geographic distance was found. Individual-based STRUCTURE analysis over 36 sites showed 7 genetic clusters. *F*_ST_ and *R*_ST_ values for ten populations reached 0.343 and 0.521, respectively thereby indicating that allele length differences are more important and contain evolutionary information. Overall, higher levels of diversity and a stronger differentiation was revealed among Iranian *P*. *pectinatus* than previously observed for temperate European regions, due to regional differences across mountain ranges over long distances.

## Introduction

Biogeographic barriers for freshwater biota can be considered at three spatial scales, namely inter and within basin and continental. At the largest spatial scale, freshwater organisms are isolated by their high mountain ranges, inability to cross oceans and vast deserts [1]. Gene flow between populations of aquatic plants is restricted by the discontinuous nature of their habitat embedded in another terrestrial landscape and as such, lakes or rivers could be considered as islands of suitable habitats. Under an Island Migration Model, increasing geographical distance between populations is expected to lead to enhanced genetic isolation, which essentially corresponds to a stepping-stone population structure and requires that dispersal is primarily local rather than long-distance [2, 3, 4, 5, 6].

Besides merely distance as a limit to connectivity, especially large deserts and mountain ranges act as additional natural barriers. The flora of the mountains provides many examples of subspecies with populations and very closely related species that show restricted gene flow due to these inherent geographic barriers [7, 8]. Isolation by distance is stronger across than alongside mountain ridges [9, 10, 11].

Drought as a climatic event is one of the most threatening challenges that occur due to climatic change and global warming [12]. The heterogeneity of habitats and environment among populations probably influence gene flow in disrupting or dispersal, resulting in isolation by environment (IBE) model in which genetic differentiation is positively correlated with environmental differentiation [13, 14]. Drought has been a problematic phenomenon for aquatic ecosystems in Mediterranean temporary pools [15] and in low rainfall regions e.g. Central and SW Asia [16] during recent years because it declines water quality and decreases aquatic plant occurrences.

The importance of submerged vegetation in aquatic ecosystems management and biodiversity has been highlighted [17, 18, 19]. Many aquatic plants have mixed modes of reproduction and different models of dispersal [20, 21]. In submerged water plants (macrophytes), sexual reproduction by seeds produces new genotypes [21]. In spite of the low clonal diversity between and within populations [20], several studies showed that aquatic plant populations often had high clonal diversity [22, 23, 24, 25] nearly similar to non-clonal plant species [26]. Among aquatic plant taxa, fennel pondweed, *Potamogeton pectinatus* L. is one of most diverse submerged aquatic species [27, 28]. This species maintains many useful physiological traits such as tolerance to a wide range of nutrients, ability to grow in oligotrophic to eutrophic waters [29], and utility to progress quality of water by removing nutrients [30]. These useful traits made the species also a potentially important biotic tool for cleaning up polluted waters by heavy metals absorption [31, 32]. Plant parts are an important source of food for many waterfowl [23].

*P*. *pectinatus* reproduces both sexually and vegetatively through tubers emerging from the rhizomes and acting as propagules. Asexual reproduction is thought to be responsible for short distance dispersal while sexual reproduction with seeds is more important to ensure long distance dispersal and long-term survival [21, 29]. There are several studies about genetic variation of *P*. *pectinatus*. Hettiarachchi & Triest [33] used isozyme markers to detect genotypes within different habitats such as freshwater and brackish water, and showed a geographic trend in genotypes among freshwater populations. Using isozyme polymorphism, Hollingsworth *et al*. [2] confirmed the importance of local clonal growth in *P*. *pectinatus*. Hangelbroek *et al* [23] used RAPD markers and showed that sexual reproduction has a very important role within a lake population. Hollingsworth *et al*. [2] also showed that isolation by distance was determined as a regulatory factor for gene flow at distances of more than 1000 km along the Atlantic and Baltic Sea coastal habitats, proposing that dispersal between populations is mainly by seeds. The effect of migrating waterfowls on plant distribution pattern and seed dispersal over large distances have been reported [22, 34, 35]. Also Mader *et al*. [22] and King *et al*. [34] who used RAPD and ISSR markers, respectively concluded that genetic distance between populations of *P*. *pectinatus* increased with geographic distance. Following the development of nine polymorphic microsatellite loci for *P*. *pectinatus* [36] an evolutionary divergence and less gene flow between two types of habitat on a regional scale [37] were shown, namely for the brackish water populations of the Baltic Sea lagoons versus freshwater inland populations of Central Europe. Genetic differentiation of *P*. *pectinatus* but also other submerged plant taxa was found between coastal brackish water and freshwater inland habitats in lowland regiosn of W. Europe by Triest *et al*. [38]. They also detected that upstream forest ponds can be detected as source populations and defections for clonal diversity to recolonize the stressful downstream river habitat. A more detailed study of the clonal diversity and fine-scaled spatial structure at individual level of *P*. *pectinatus* in river populations and managed pond [39] revealed that the ponds populations had a higher amount of gene and clonal diversity than those of rivers. Han *et al*. [40] using AFLP showed that *P*. *pectinatus* maintained a high level of variation within and between two contrasting and distant lakes in China. Those previous studies over the last decades confirmed that *P*. *pectinatus* has a mixed reproduction system, an overall high level of gene diversity and an increased differentiation over long distances of hundreds of kilometers. All previous studies were done in temperate and temperate-cold areas having many permanent water bodies and often at close vicinity. Therefore, it can be hypothesized that *P*. *pectinatus* populations might exhibit a stronger genetic differentiation over similar distances across mountain ranges and in a drier climate zone with often temporary habitats of very isolated wetlands areas and rivers habitats that show strong fluctuations due to high evaporation levels and use for irrigation in agriculture.

The specific biogeographic location of Iran [41] with its several large mountain ranges and deserts present a totally different suite of habitat types at a presumed low connectivity. The Alborz mountain ridge is located in the north whereas the Zagros mountain region is extending from northwest to southeast of Iran. The mountains of Kerman are located at the eastern edge of Zagros. Dashte Kavir is a desert region in the center of Iran. These geographic characteristics are assumed to create a differentiation and isolation between north and south populations and thus can pose an additional barrier to dispersal besides the distance to overcome. Along the same side of mountain ranges, these biogeographic barriers promote homogenization of aquatic biota [42]. Additionally, the mountains of Kerman located in the southeast of Iran are a potential barrier between north and south populations. Historically, these barriers identified as dominant factor in determining the composition of regional organisms [43, 44]. The world’s second largest saline lake, Urmieh lake, is located in the north west region. Iran has a great diversity of aquatic ecosystems many of which have been recorded in the Ramsar Convention [45]. The largest portion of freshwater bodies in Iran is composed of many large rivers and lagoons. Different challenges such as the effects of human-based threats disrupt their ecological characteristics [46].

Our aim was to test, by considering sites with *P*. *pectinatus* populations over a long gradient of regions across mountain ranges as potential barriers, whether in such habitats

1. *P*. *pectinatus* can maintain its usually high levels of allele and gene diversity.
2. Differentiation over distances and across mountain ranges and around deserts would be more pronounced and structured than in temperate regions.

Therefore we considered a sampling design with a large number of sites across the various biogeographic regions and used the same microsatellite loci as a previous European studies, following a preliminary taxon verification with nuclear ITS and maternal chloroplast marker genes.

## Materials and Methods

### The species

Fennel pondweed, *P*. *pectinatus* L. (= *Stuckenia pectinata*, see Lindqvist *et al*. [47] for taxonomic revision) is a submerged aquatic plant with a nearly cosmopolitan distribution ranging from sub-arctic to tropic regions, of different types of waterbodies and adapted to a large variation in water depth, flow regime, trophic status and salinity [48,37]. *Potamogeton pectinatus* reproduces both sexually and vegetatively through propagules emerging from the rhizomes [29]. It is an important source of food for many waterfowls [23]. This species harbors many useful physiological traits such as tolerance to a wide range of nutrients, capability to grow in oligotrophic to eutrophic waters [29], and ability to improve quality of water by removing nutrients [30]. These useful traits made the species a potentially important candidate for cleaning up polluted waters by heavy metals absorption [31, 32].

### Study area and sampling design

**Fig 1:**
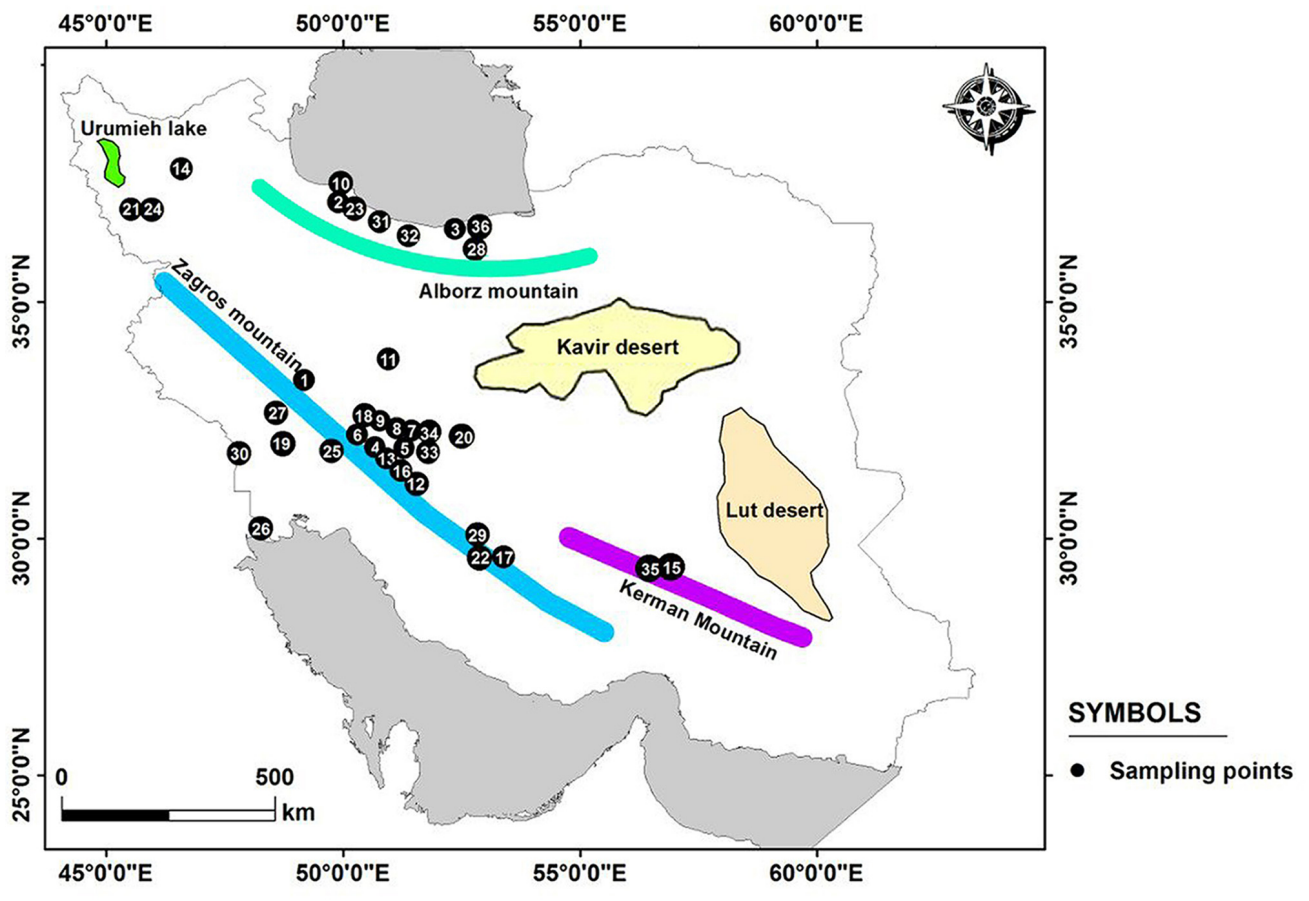
Study area indicating 36 sites of *Potamogeton pectinatus* in Iran. **Numbering as in** Table 1. 1: Aligoodarz; 2: Amirkelaieh; 3: Azbaran; 4: Barm; 5: Borujen; 6: Chaghakhor; 7: Chamaseman, 8: Chamgordan; 9: Chamtagh; 10: Dahaneh sefidrood; 11: Delijan; 12: Dizicheh; 13: Gandoman; 14: Ghoorigol; 15:Googhar; 16: Hamzeali; 17: Hasanabad; 18: Hojatabad; 19: Izeh; 20: Jarghoieh; 21: Kaniborazan; 22: Komjan; 23: Langrood; 24: Miandoab; 25: Miangaran; 26: Shadegan; 27: Shushtar; 28:Siahrood; 29: Sivand; 30: Soosangerd; 31: Tonekabon; 32: Valasht; 33: Varnamkhast; 34: Vimcheh; 35: Yaschaman; 36: Zaghmarz.

*P*. *pectinatus* plants were collected in 2015–2016 in thirty six sites from wetlands, lakes and rivers in Iran (Table 1, Fig 1). No specific permission was required for these locations/activities and field studies did not involved endangered or protected species. The geographic distance between pairs of populations ranged from 20 to 1200 km. Because the populations of this species are still declining due to human activities and recent drought in Iran; in each site we collected 1–20 individual shoots (ramets) at 2–3 m intervals. Design was to cover the entire distribution range and diversity of aquatic habitats within Iran. Our samples usually were in the main channel in downstream parts of rivers with low speed.

**Table 1:** Regions situation, location details and features of *P*. *pectinatus* populations in Iran, their nuclear ITS, trnH psbA haplotype length, voucher number and type of water body and a diagnostic morphological feature.

Locality	Region	Long (E)	Lat (N)	Nuclear ITS	trnH-psbA Haplotype	Voucher	Water body	Mean Rank*
**1-Aligoodarz**	N. Zagros	49.165	33.478	ITS-B	380	20157	River	1.75
**2-Amirkelaieh**	N. Alborz	50.222	37.277	ITS-A	379	20147	Wetland	0.25
**3-Azbaran**	N. Alborz	52.476	36.650	ITS-A	377	20152	Wetland	1.11
**4-Barm**	N. Zagros	51.208	31.577	ITS-A	378	20174	Spring	1.12
**5-Boroujen**	N. Zagros	51.327	31.959	ITS-B	380	20168	Stream	1.12
**6-Chaghakhor**	N. Zagros	50.932	31.923	ITS-A	377	20169	Wetland	1.03
**7-Chamaseman**	N. Zagros	51.223	32.3727	ITS-A	380	20164	Reservoir	0.58
**8-Chamgordan**	N. Zagros	51.342	32.385	ITS-A	379	20160	River	0.99
**9-Chamtagh**	N. Zagros	50.993	32.459	ITS-A	379	20159	River	0.47
**10-Dahaneh sefidrood**	N. Alborz	50.181	37.383	ITS-A	378	20146	River	0.52
**11-Delijan**	N. Zagros	50.682	33.990	ITS-A	378	20156	Stream	1.19
**12-Dizicheh**	N. Zagros	51.527	32.376	ITS-A	379	20162	River	0.33
**13-Gandoman**	N. Zagros	51.084	31.814	ITS-A	377	20172	Wetland	0.5
**14-Ghoorigol**	W. Alborz	46.705	37.915	ITS-A	378	20145	Wetland	1.15
**15-Googhar**	Kerman Ms	56.637	29.483	ITS-B	380	20179	River	3.11
**16-Hamzeali**	N. Zagros	51.076	31.835	ITS-A	377	20171	Canal	1.14
**17-Hasanabad**	SE. Zagros	53.336	29.656	ITS-A	378	20178	River	0.54
**18-Hojatabad**	N. Zagros	50.842	32.510	ITS-A	379	20158	River	1.12
**19-Izeh**	SW. Zagros	48.861	32.051	ITS-A	379	20167	River	1.18
**20-Jarghoieh**	N. Zagros	52.617	32.162	ITS-A	379	20165	Wetland	0.55
**21-Kaniborazan**	W. Alborz	45.783	36.966	ITS-A	378	20150	Wetland	1.27
**22-Komjan**	SE. Zagros	53.148	29.669	ITS-A	378	20177	River	0.85
**23-Langrood**	N. Alborz	50.162	37.187	ITS-A	378	20148	River	0.29
**24-Miandoab**	W. Alborz	46.089	36.990	ITS-A	378	20149	Wetland	1.11
**25-Miangaran**	SW. Zagros	49.870	31.882	ITS-A	379	20170	Wetland	0.28
**26-Shadegan**	SW. Zagros	48.330	30.266	ITS-A	378	20175	Wetland	1.23
**27-Shushtar**	SW. Zagros	48.859	32.051	ITS-A	378	20166	River	0.75
**28-Siahrood**	N. Alborz	52.885	36.303	ITS-A	378	20155	River	0.9
**29-Sivand**	SE. Zagros	52.923	30.113	ITS-A	378	20176	River	0.96
**30-Soosangerd**	SW. Zagros	47.916	31.795	ITS-A	378	20173	River	1.2
**31-Tonekabon**	N. Alborz	50.900	36.783	ITS-A	378	20151	River	0.89
**32-Valasht**	N. Alborz	51.541	36.454	ITS-A	378	20154	Lake	0.89
**33-Varnamkhast**	N. Zagros	51.367	32.377	ITS-A	379	20161	River	1.22
**34-Vimcheh**	N. Zagros	51.484	32.374	ITS-A	379	20163	River	0.76
**35-Yaschaman**	Kerman Ms	56.624	29.454	ITS-B	380	20180	Stream	2.2
**36-Zaghmarz**	N. Alborz	52.891	36.488	ITS-A	378	20153	Wetland	1.03

*: Mean Rank compute by Kruskal-Wallis

Samples were dried on silica gel prior to extraction and a voucher specimen for each population was deposited at HUI (Herbarium of the University of Isfahan). One subset (one individual from one sampling site) of the collection was used for cpDNA haplotypes and ITS (accession number: DQ840279.1) characterization (results not shown in this paper). Identification of *P*. *pectinatus* populations was based on cpDNA sequence variability in trnH-psbA referring to *P*. *pectinatus* haplotypes and on two nuclear spacers (ITS1 and ITS2)—as in Triest and Sierens [49]. A more detailed study of genetic diversity and structure at the population level was done on a subset of 10 populations where a sufficient large number of different genets could be considered.

For each specimen we measured 29 morphological characters. Among these, the leaf apex and leaf width (36 measurements per site) could differentiate morphologically the specimens characterized for a nuclear ITS-A or B. B. Mean rank for Krustal-Wallis was between 1.12–3.11 mm for the group of specimens sharing ITS-B. Morphological measurements were tested for significant differences with one-way ANOVA (Kruskal-Wallis) and pairwise Mann-Whitney U test.

### DNA extraction and microsatellite amplification

Genomic DNA was extracted from leaf tissue using CTAB by Gawel and Jarret [50] with some modifications. All individuals were genotyped at nine nuclear microsatellite loci:

Potpect24, Potpect26, Potpect28, Potpect32, Potpect34, Potpect37, Potpect39, Potpect40, and Potpect42 [36]. Amplification of these nine loci was not performed in three multiplex PCR as described in Nies & Reusch [36], but into a unique multiplex PCR. This single amplification was made possible by the use of the QIAGEN Multiplex PCR Kit (QIAGEN) in a final volume of 10.5 μL, as follow: 25 ng of DNA template, 5 μL 2× QIAGEN Multiplex PCR Master Mix [QIAGEN Multiplex PCR Buffer, pH 8.7, containing dNTPs, QIAGEN HotStar Taq DNA Polymerase, and 6 mM MgCl2 (for a final concentration of 3 mM)], 1 μL Q-Solution (59 concentrated proprietary QIAGEN PCR additive), 1 μL of a primer mix with 2 μM of each primer (for a 0.2 μM final concentration of each primer) and 1 μL of highly pure water obtained from a Milli-Q Synthesis A10 (Millipore, Molsheim, France). PCR were carried out in 96-well plates on a MyCycler TM thermal cycler (BIO-RAD) under the following conditions: 15 min denaturing at 95'C, [3000 denaturing at 94'C, 1.5 min annealing at 57'C and 1 min extension at 72'C] × 30 cycles and a final extension step at 72'C for 10 min. PCR were carried out in 96-well plates on a MyCycler TM thermal cycler (BIO-RAD) under the following conditions: 4 min denaturing at 94'C, [3000 denaturing at 94'C, 1 min annealing at 57'C and 1 min extension at 72'C] × 30 cycles and a final extension step at 72'C for 30 min. PCR products were run on ABI3730XL sequencer (Macrogen, Seoul, Korea) and fragments were scored with GeneMarker V2.20 (SoftGenetics LLC, State College, USA). Although *P*. *pectinatus* is hexaploid, the identified primer pairs amplify microsatellite loci that are ‘‘diploids”, confirming the observations of Nies & Reusch [36, 37]. To explain this phenomenon, these authors support the idea that the time that has elapsed after the polyploidisation event was sufficient to cause genetic divergence of the microsatellites on the different sets of homologous chromosomes [37]. After excluding forty eight repeated multilocus genotypes (MLGs) from the data set, we calculated genetic diversity measures on 133 individuals using FSTAT 2.9.3 [51] and GenAlex 6.5 [52]. At locus level, we estimated the number of alleles (A), effective number of alleles (Ae), observed heterozygosity (Ho), expected and unbiased expected heterozygosity (He and uHe), Nei’s heterozygosity at subpopulation level (Hs) and total (Ht), Wright’s F statistics [53] with inbreeding coefficient (*F*_IS_), total inbreeding (*F*_IT_), subpopulation differentiation (*F*_ST_) and gene flow (Nm) using GenAlex. Weir & Cockerham [54] estimation of *F*_IT_ (CapF), *F*_IS_ (small f) and *F*_ST_ (Theta) following Rousset [55] were calculated with FSTAT. Inbreeding values for the total populations are given by small f and jackknifed over loci (FSTAT).

We tested for recent bottlenecks in each population under the two-phase model (TPM) with 95% single-step mutations and 5% multiple-step mutations (Wilcoxon’s test 1-tailed) using bottleneck 1.2.02 [56]. Genetic structure was assessed with a three-level analysis of molecular variance (AMOVA) [57] using hierarchical and standardized fixation indices for 5 regions, 10 populations and 68 individuals (GenAlex). Pairwise FST-values were calculated between all pairs of populations and tested for significant differentiation using 999 permutations. Isolation-by-distance between pairs of populations and their geographical distances was tested with ϴ/1- ϴ considering straight flight distances, log transformed [55] between populations in a Mantel test using 1000 randomizations [58]. An estimator of actual differentiation *D*_est_ [59] was calculated between all pairs of populations using SMOGD [60] and used in a Mantel test with log transformed straight flight distances. To infer the population structure on basis of assignment of individual genotypes into groups, a Bayesian clustering method [61] was carried out using STRUCTURE version 2.3. We tested for the number of clusters (K) in ten independent runs from 1 to 14 (10,000 burn-in, 10,000 Markov chain Monte Carlo replicates in each run), without using sampling site as a prior to assess convergence of the estimated ln probability of data, ln (PD). Runs were carried out assuming admixture and an independent model of allele frequencies. The number of clusters was determined from the K with the highest posterior probability and using the second-order rate of change of the likelihood function ΔK, as suggested by Evanno *et al*. [62]. For testing the evidence of scoring error, Evidence of large allele dropout and Evidence of null allele we used Micro-checker 2.2 software. Also we used POPTREE2 [63] software for cluster analysis. Isolation by distance and R_ST_ was tested by calculating multilocus estimates of kinship coefficient (Fij) between all pairs of individuals implemented in the software SPAGeDi v1.4 [64].

## Results

The haplotypes obtained from amplified product lengths of TrnH-PsbA showed congruent differences for Aligoodarz (1), Borujen (5), Googhar (15) and Yaschaman (35) when compared to all other populations. Morphological measurements between this group (mean rank of 1.12–3.11 mm) and specimens from other sites (mean rank of 0.25–1.27 mm) were significant with one-way ANOVA (Kruskal-Wallis) (P = 0.036) and pairwise Mann-Whitney U test (P = 0.012). The lowest morphological value was 0.25 mm (2-Amirkelaieh) whereas the highest value 3.11 mm (15-Googhar).

### Microsatellite loci properties

As the primers were previously developed and used on European *Potamogeton pectinatus* populations, we tested these cross-amplified loci with microchecker, but there was no evidence for null alleles, large allele dropout or scoring error due to stuttering. Over all sites and loci, the nine microsatellite loci revealed a total of 130 alleles. The mean number of alleles was highest for potpec 39 (3.3) and lowest for potpec24 (1.9) with an overall observed heterozygosity (Ho = 0.2–0.7) nearly similar to the overall expected heterozygosity (He = 0.3–0.7) (Table 2).

**Table 2:** Features of 9 microsatellite loci for *Potamogeton pectinatus* as observed in 36 sites of this study Total number of alleles (A), overall observed heteroygosity (H_o_) and overall expected unbiased heterozygosity (uH_E_), are given for 36 sites.

Locus	Primer sequence	Repeat motif	Range	A	H_o_	uH_E_	Gen Bank accession no.
**Potpect 24**	F Ned- TCAGTGAAAGAAAGCCAGGA	(GA)n	132–186	1.9	0.210	0.314	AY568087
	R GGGCTTATGGCGTTATCAA						
**Potpect 26**	**F** Fam-GTATAGGCGAGGTGCGAGAG	(CT)*n*	234–250	2.4	0.595	0.541	AY568088
	**R** CTTCATGTCGACCACCTTCC						
**Potpect 28**	**F** Fam-TCGTTTCCTCCATTCGTAGG	(GA)*n*	164–187	2.5	0.684	0.560	AY568089
	**R** AATAAAAAGGGCCCAGACC						
**Potpect 32**	**F** Hex-CAGCAAACGAAACAACCAAA	(GA)*n*	202–247	2.3	0.251	0.433	AY568090
	**R** AAAAGAAGCCGTTGTTTACAGAG						
**Potpect 34**	**F** Fam-GTAAGGCAAGCAGCGTCAAC	(GA)*n*	223–251	3.0	0.612	0.639	AY568091
	**R** GTTTGTGAGCTAGCGGGAAG						
**Potpect 37**	**F** Hex-CACTTCCTCTGTGCTGCTTG	(CT)*n*	137–177	2.6	0.609	0.614	AY568092
	**R** GCGTGCTCTTCCTGAGTTCT						
**Potpect 39**	**F** Hex-TCACAACACCTCACCCAGAA	(GA)*n*	212–246	3.3	0.621	0.629	AY568093
	**R** CCATTTCCATTCCTCACTGC						
**Potpect 40**	**F** Ned-AAATCTCCAAATATTTCCACTGTTG	(GA)*n*	179–208	2.5	0.556	0.542	AY568094
	**R** CAAAGATTGAGCTCCCCAAA						
**Potpect 42**	**F** Ned-TTAGCAAGTGGGTGGGTTTC	(CT)*n*	177–208	3.1	0.761	0.706	AY568095
	**R** TGCACTCGTGTGTCTCTTCC						

Out of 181 ramets in 36 sites, 133 genets (MLG) were obtained with re-encounters only occurring within a same site Table 3). Despite the small number of ramets available per site (2–15), the obtained number of genets remained between 2–11, thereby giving a range of values for clonal richness from 0–1 (Table 3). The mean number of alleles varied from 1.3–5.2, the effective number of alleles from 1.5–4.0. Considering these 36 sites, their number of private alleles ranged between 0–7, the observed heterozygosity between 0.2–0.7, unbiased expected heterozygosity from 0.111–0.815. For ten populations, allelic richness was between 1.5–3.6 whereas the inbreeding coefficient *F*_IS_ was estimated -0.077–0.846. The populations of Azbaran (3), Vimcheh (34) and Izeh (19) showed evidence of recent bottleneck events (Table 3).

**Table 3:** Clonal diversity (N, G, R) and gene diversity measures of *Potamogeton pectinatus* individuals at 36 sites with mean number of alleles (Na), effective number of alleles (Ae), number of private alleles (PA), observed heterozygosity (Ho), unbiased expected heterozygosity (uHe); and for 10 sites considered as populations the inbreeding coefficient (*F*_IS_) Bottleneck test and allelic richness (Ar).

Pop code	No ramets (N)	No genets (G)	Clonal richness (R)	Na	Ae	PA	Ho	uHE	*F*_IS_	Bottleneck (TPM) Wilcoxon	Ar
**1-Aligoodarz**	7	2	0.2	1.8	1.7	1	0.333	0.463	NA	NA	NA
**2-Amirkelaieh**	10	8	0.7	4.4	2.9	2	0.664	0.644	0.021	0.450	3.2
**3-Azbaran**	10	4	0.3	1.9	1.8	0	0.667	0.414	0.846*	0.004*	1.9
**4-Barm**	3	3	1	3.4	2.9	0	0.593	0.756	NA	NA	NA
**5-Borujen**	2	2	1	1.3	1.3	0	0.111	0.111	NA	NA	NA
**6-Chaghakhor**	4	3	0.7	1.9	1.7	1	0.296	0.385	NA	NA	NA
**7-Chamaseman**	3	3	1	2.0	1.6	0	0.296	0.430	NA	NA	NA
**8-Chamgordan**	2	2	1	1.7	1.7	2	0.667	0.519	NA	NA	NA
**9-Chamtagh**	2	2	1	2.4	2.2	1	0.389	0.667	NA	NA	NA
**10-Dahaneh sefidrood**	3	2	0.5	2.2	2.0	0	0.556	0.537	NA	NA	NA
**11-Delijan**	4	4	1	3.2	2.7	2	0.778	0.679	NA	NA	NA
**12-Dizicheh**	2	2	1	3.0	2.8	0	0.611	0.815	NA	NA	NA
**13-Gandoman**	2	2	1	2.6	2.3	1	0.500	0.630	NA	NA	NA
**14-Ghoorigol**	6	3	0.4	1.8	1.7	1	0.704	0.452	NA	NA	NA
**15-Ghooghar**	3	3	1	1.7	1.5	0	0.241	0.300	NA	NA	NA
**16-Hamzeali**	2	2	1	1.1	1.1	0	0.111	0.111	NA	NA	NA
**17-Hasanabad**	2	1	0.0	1.7	1.7	0	0.667	0.667	NA	NA	NA
**18-Hojatabad**	2	2	1	1.7	1.6	0	0.500	0.407	NA	NA	NA
**19-Izeh**	14	6	0.4	4.4	3.5	1	0.593	0.709	-0.077	0.007*	3.4
**20-Jarghoieh**	4	4	1	3.2	2.6	1	0.472	0.567	NA	NA	NA
**21-Kaniborazan**	15	11	0.7	3.9	2.9	7	0.763	0.619	0.177*	0.069	2.8
**22-Komjan**	5	5	1	2.7	2.5	1	0.717	0.574	0.217*	0.349	2.5
**23-Langrood**	5	3	0.5	2.7	2.5	0	0.630	0.556	NA	NA	NA
**24-Miandoab**	6	6	1	4.2	3.1	5	0.494	0.618	-0.269*	0.307	2.6
**25-Miangaran**	3	2	0.5	1.7	1.6	0	0.611	0.426	NA	NA	NA
**26-Shadegan**	3	2	0.5	1.7	1.5	0	0.444	0.389	NA	NA	NA
**27-Shushtar**	11	8	0.7	5.2	4.0	3	0.679	0.741	0.183*	0.477	3.6
**28-Siahrood**	5	2	0.2	2.9	2.7	0	0.778	0.778	NA	NA	NA
**29-Sivand**	5	3	0.5	3.1	2.7	0	0.796	0.737	NA	NA	NA
**30-Soosangerd**	5	5	1	3.3	2.8	2	0.683	0.639	0.296*	0.222	2.9
**31-Tonekabon**	2	1	0.0	1.8	1.8	0	0.778	0.778	NA	NA	NA
**32-Valasht**	7	4	0.5	2.8	2.3	0	0.648	0.525	NA	NA	NA
**33-Varnamkhast**	4	3	0.7	3.1	2.8	2	0.389	0.737	NA	NA	NA
**34-Vimcheh**	6	6	1	3.8	2.1	2	0.433	0.521	-0.299*	0.006*	2.6
**35-Yaschaman**	9	9	1	2.8	1.6	0	0.228	0.311	0.091	0.059	1.5
**36-Zaghmarz**	3	3	1	3.0	2.7	0	0.778	0.704	NA	NA	NA
**Mean/total**	5	3.7	0.7	2.6	2.2	0.97	0.54	0.55			

* indicates populations with significant *F*_IS_ (p <0.05).

From the 10 considered populations, the percent of individuals assigned to the same population was higher (76%) than those assigned to other populations (24%). The populations of Amirkelaieh (2), Azbaran (3) and Kaniborazan (21) did not share any allele.

**Fig 2:**
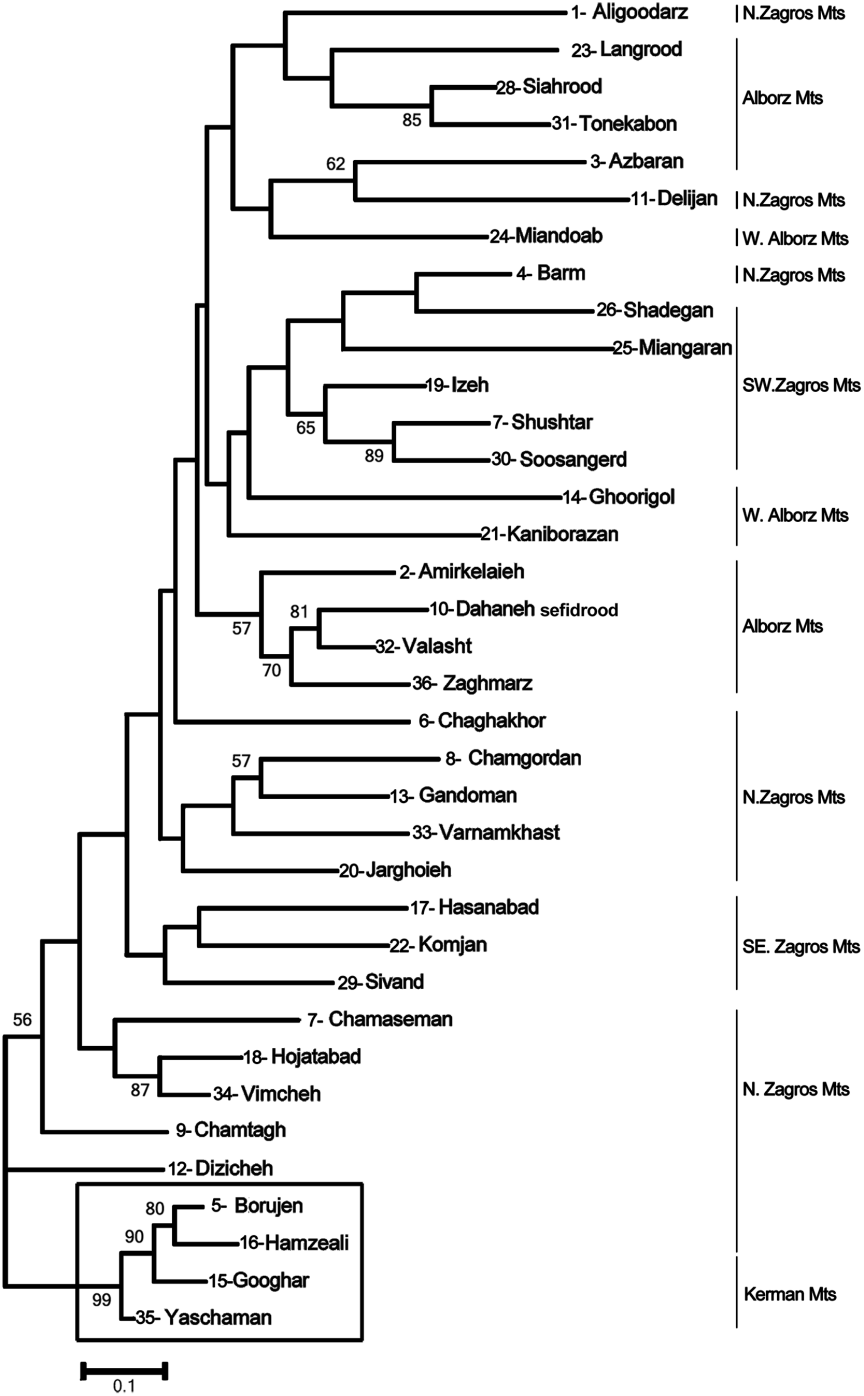
Neighbor joining tree of microsatellite diversity in 36 sites of *P*. *pectinatus*.

A Neighbor Joining tree of microsatellite gene diversity was congruent with the *P*. *pectinatus* identity of nuclear ITS and trnH-psbA (380 bp length) for the samples from Hamzeali (16) Borujen (5), Googhar (15), and Yaschaman (36) (Fig 2).

**Fig 3:**
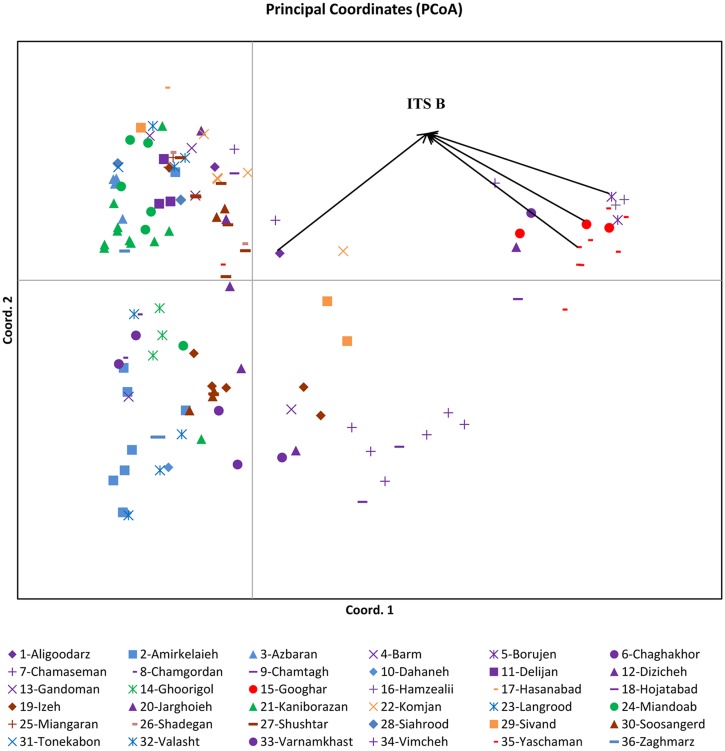
PCoA grouping of 133 individuals from 36 sites. The samples from Aligoodarz (1), Borujen (5), Googhar (15), and Yaschaman (36), Hamzeali (16) are grouped separately (ITS B). (symbols of sites situated along the same mountain range share a same color).

**Fig 4:**
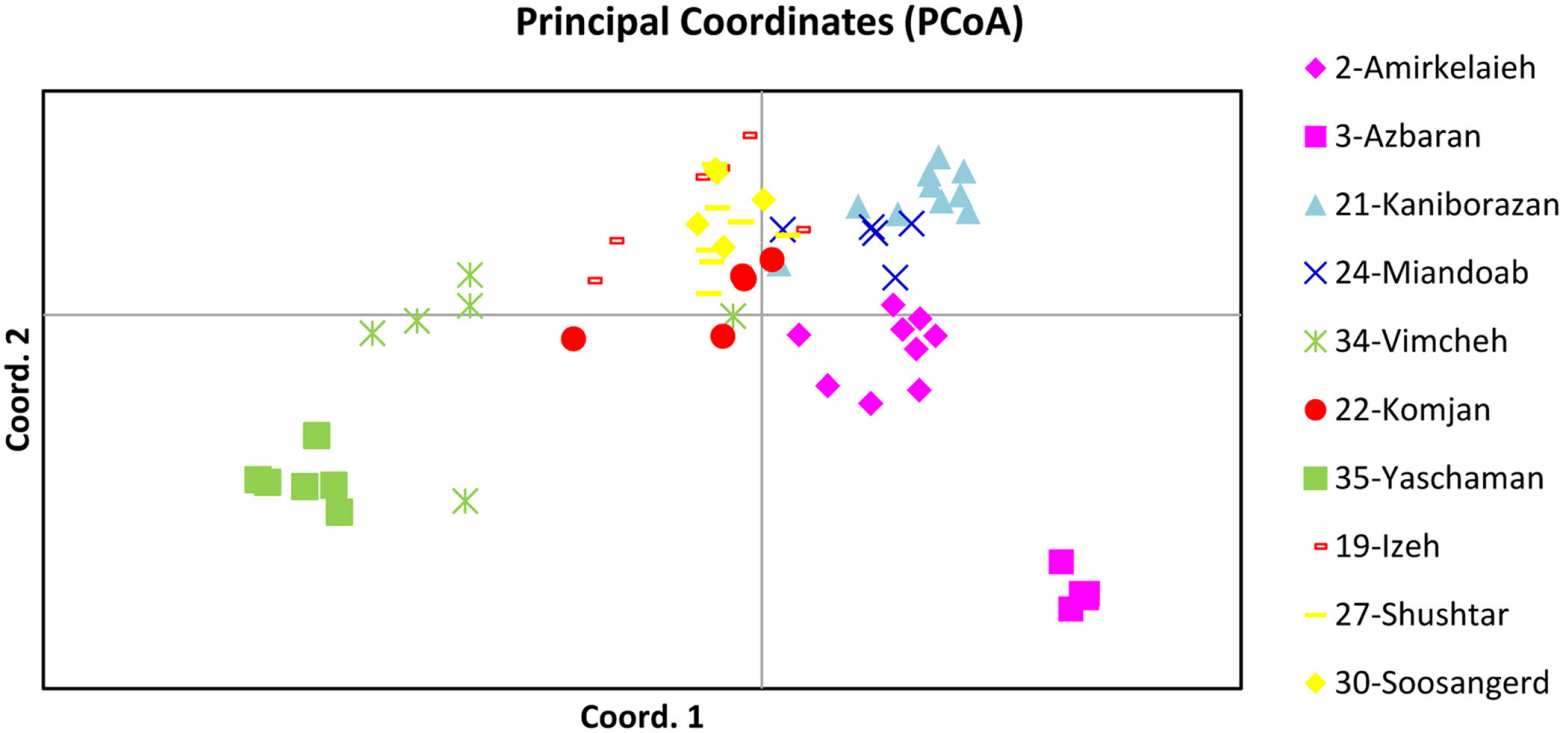
PCoA of 10 populations from each major region.

A PCoA at the level of all individuals revealed a gradient along a first axis that explained 28.9% of all variation but where several individuals were clearly separated as a group (Figs 3 and 4). Most of these individuals were also tested for ITS and showed the ITS-B sequence. A PCoA confined to ten populations that explained 28.8% of all variation revealed a very clear separation of most populations from each other. A gradient can be observed from individuals of N. Alborz, around lake Urumieh, S.E. Zagros, N. Zagros and SE Zagros to Kerman mountain ranges.

Percentage of variation explained by the first three axes in PCoA at 10 populations is 28.80% and 17.94% for first and second axes, respectively.

The range of *F*_ST_ was between 0.156–0.595 among the ten populations. The lowest *F*_ST_ was between Izeh (19) and Amirkelaieh (2) and the highest was between Azbaran (3) and Yaschaman (35) (Table 4).

**Table 4:** Pairwise Population significance *F*_ST_ (below diagonal) and *D*_est_ (above diagonal) values.

Amirkelaieh	Azbaran	Izeh	Kaniborazan	Komjan	Miandoab	Shushtar	Soosangerd	Vimcheh	Yaschamn	
-	0.253	0.374	0.513	0.735	0.340	0.607	0.439	0.467	0.699	Amirkelaieh
**0.301**	-	0.799	0.675	0.775	0.621	0.829	0.779	0.875	0.925	Azbaran
**0.156**	0.422	-	0.461	0.450	0.474	0.084	0.012	0.216	0.440	Izeh
**0.250**	0.419	0.201	-	0.657	0.538	0.679	0.622	0.679	0.864	Kaniborazan
**0.299**	0.485	0.233	0.307	-	0.636	0.336	0.566	0.347	0.478	Komjan
**0.240**	0.401	0.235	0.287	0.298	-	0.629	0.476	0.645	0.779	Miandoab
**0.197**	0.385	-	0.263	0.206	0.241	-	0.004	0.461	0.536	Shushtar
**0.196**	0.456	-	0.293	0.303	0.266	0.008	-	0.356	0.511	Soosangerd
**0.258**	0.516	0.135	0.348	0.248	0.339	0.208	0.238	-	0.103	Vimcheh
**0.404**	0.595	0.312	0.447	0.415	0.461	0.340	0.398	0.207	-	Yaschaman

**Fig 5:**
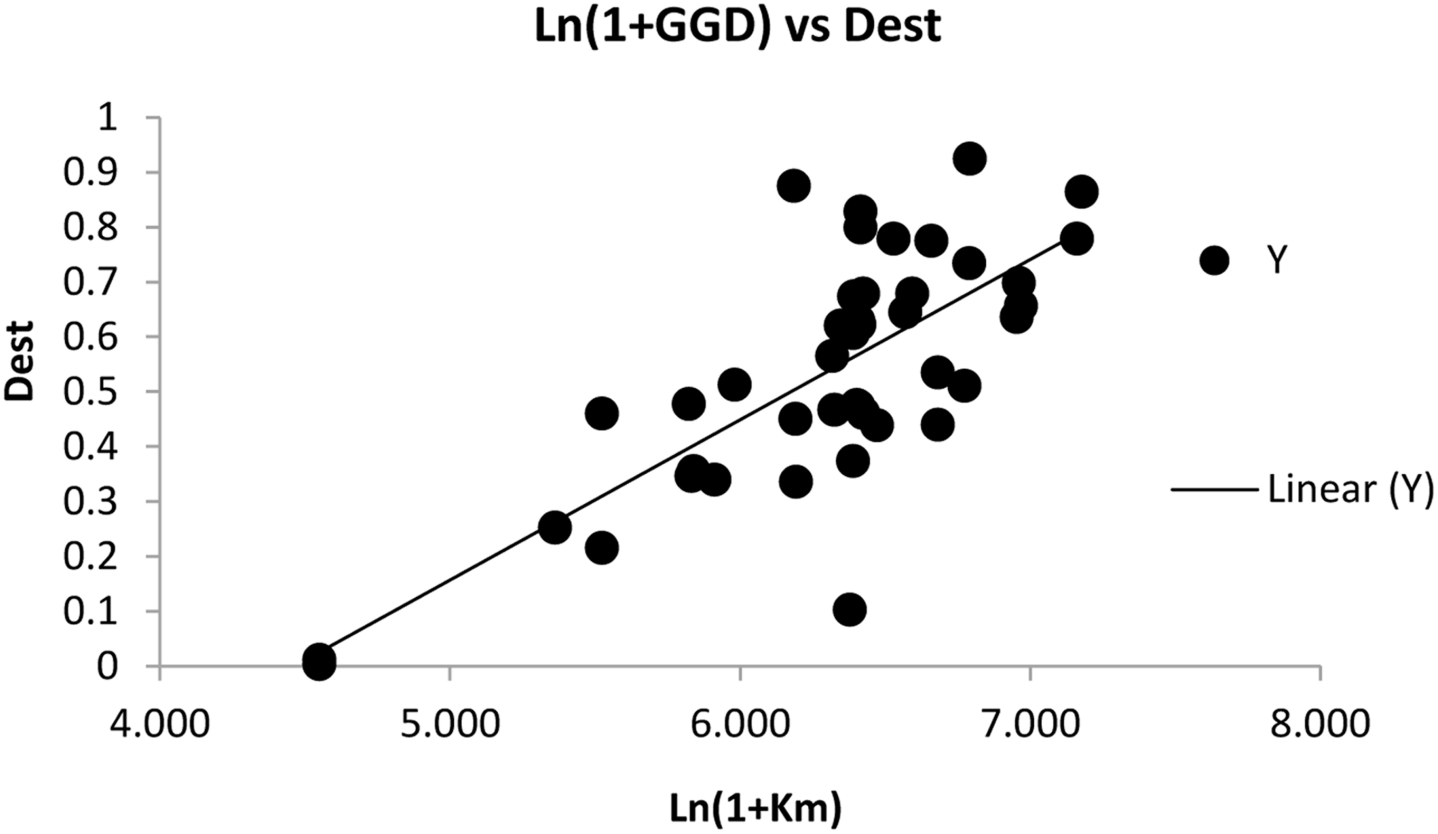
Relationship between *D*_est_ and geographic distance Ln(1+km) at population level.

The range of *D*_est_ values were between from 0.001 to 0.925. There was no relationship at all between Fst and *D*_est_ (Pearson correlation = 0.007) indicating that allelic differentiation can be more important as an explanatory variable of IBD. There was a positive relationship between *F*_ST_ and geographic distance (R^2^ = 0.35 and P = 0.01) but there was an improved positive correlation between *D*_est_ and Ln (1+Km) (P = 0.01, R^2^ = 0.56) (Fig 5).

An AMOVA showed that the percentage of molecular variance among regions and populations were 15% and 18%, respectively (Table 5).

**Table 5:** AMOVA result for 6 mountainous regions.

**Source**	**df**	**SS**	**MS**	**Est. Var.**	**%**
**Among Regions**	5	126.974	25.395	0.609	15%
**Among Pops**	4	50.167	12.542	0.725	18%
**Among Indiv**	58	152.654	2.632	0.000	0%
**Within Indiv**	68	179.000	2.632	2.632	66%
**Total**	135	508.794		3.966	100%
**F-Statistics**	**Value**	**P(rand > = data)**			
**Frt**	0.153	0.001			
**Fsr**	0.216	0.001			
**Fst**	0.336	0.001			
**Fis**	0.000	0.493			
**Fit**	0.336	0.001			
Frt max	0.259				
F'rt	0.593				

**Fig 6:**
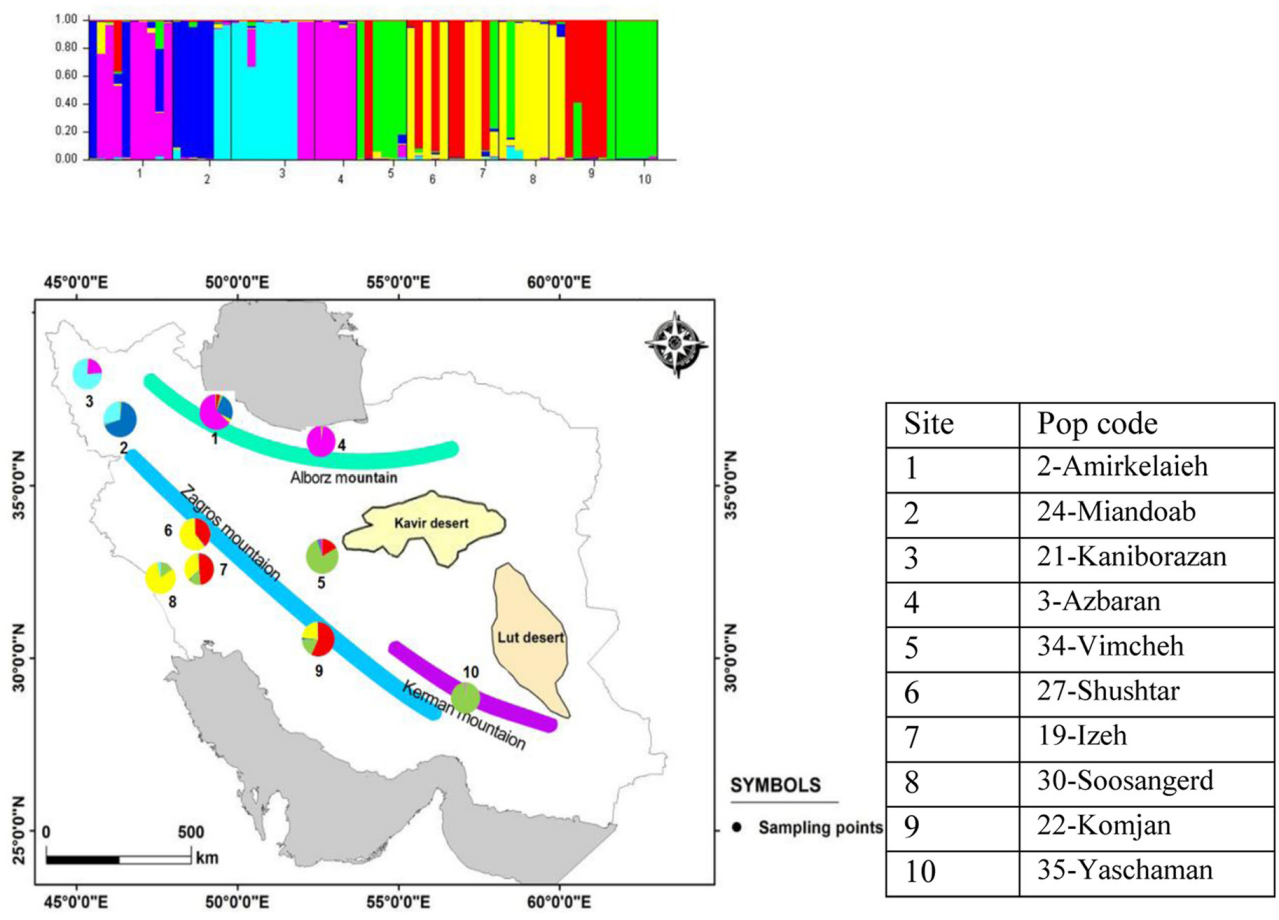
Distribution map of genetic structure of 10 populations of *Potamogeton pectinatus* in Iran.

The genetic structure of 10 populations of *Potamogeton pectinatus* in Iran showed 6 genetic clusters (Fig 6). The samples from Yaschaman (35) are placed separately.

Genetic analyses for 10 populations at 5 distance classes showed that *F*_ST_ and *R*_ST_ values were 0.343 and 0.485, respectively. Table 6 indicated special genetic structure analysis for these classes.

**Table 6:** Spatial genetic structure analysis for 5 distance classes (using 10 populations).

Dist classes	1	2	3	4	5
**Max distance**	340	570	610	796	1308
**Mean distance**	178	472	600	687	1022
***F***_**ST**_	0.2174*	0.3254	0.3154	0.3629	0.4958**
***R***_**ST**_	0.3202*	0.3293*	0.4955	0.5818**	0.6975**

* are significant values lower than average;

** are significant values higher than average.

The higher values for *R*_ST_ indicated that allele length differences are more important over the entire study area and evolutionary significant through low historical connectivity. In the second distance class, *F*_ST_ and *R*_ST_ had a similar value (0.32–0.33). In the largest distance class, the *R*_ST_ value (0.698) and *F*_ST_ value (0.496) were highest. Also in all distance classes, except second distance class, the values for *R*_ST_ were significantly higher than for *F*_ST_. *F*_ST_ for all loci P(1-sided test, H1: obs>exp) between neighboring populations of Izeh and Shushtar was not significant. Genetic analyses at the individual level in 5 distance classes showed that pairwise correlation coefficients of allele size are significant for upper distance classes (beyond 610 km).

## Discussion

### Allele and gene diversity

In spite of preliminary studies on the vegetative mode of reproduction of *P*. *pectinatus* and previous genetic studies on submerged aquatic plants based on dominant markers [29, 33, 2], as well as in other studies on co-dominant markers [36, 39] a high level of allelic and gene diversity was shown. Several studies showed that aquatic plant populations often had high clonal diversity [22, 23, 24, 25] nearly similar to non-clonal plant species [26]. Among aquatic plant taxa, fennel pondweed, *P*. *pectinatus* L. is one of most diverse submerged aquatic species [27, 28]. Across the wide study area we observed 130 different alleles for 133 individuals whereas Triest *et al* [39] and Nies & Reusch [36] observed 56 and 65 alleles with microsatellites, respectively in a much larger amount of individuals. This can be explained from the geographic distances that reached only up to 250 km whereas our study covered sites at distance up to1200 km, potentially leading to detection of much more allelic variants and a greater isolation by distance between populations. The lower number of samples per site clearly allowed to obtain many more alleles than in temperate regions and to detect relevant genetic structures in agreement with biogeography and mountain ranges, the sample size did not prevent us from observing the strong differentiation (with more samples/site, alleles would remain different across sites and regions). Other researchers also used the low sample size for example Rodriguez-Bonilla *et al* [65] in genetic diversity evaluation of sweet potato in Puerto Rico used 137 landraces, Population Genetics study of the Sao Tome Caecilian (Gymnophiona: Dermophiidae: Schistometopum thomense) revealed strong geographic structuring with 138 specimens by Stoeltin *et al* [66], Jackrel and Wootton [67] used low sample size in assessment variation of riparian plants within and among species shapes river communities.

Under an Island Migration Model, increasing geographical distance among populations is expected to lead to enhanced genetic isolation, which essentially corresponds to a stepping-stone population structure and requires that dispersal is primarily local rather than long-distance [2, 3,4,5,6]. Maximum geographic distance can have a strong effect on among population diversity [68]. With regard to genetic differentiation, observed *F*_ST_ value in this study was 0.5 that was relatively high due to spatial differences of studied populations and stochastic founder events as in Triest *et al* [39] and Nies & Reusch [36]. However, the allelic differentiation (*D*_est_) was much more pronounced because of the large amount of alleles detected across the different areas. Also the He value in our study is higher than in central or western Europe [36, 39]. Genotypic variation and recombination in *P*. *pectinatus* due to sexual reproduction is most likely explaining the observed high genetic diversity [22, 23]. The mixed mode of reproduction in *P*. *pectinatus* has an important effect on its genetic structure [20] and we also observed some clonal repeats among ramets within a site. *P*. *pectinatus* is a polymorphic species with high phenotypic plasticity [28]. As a phenotypic plasticity phenomenon, the plants may produce more seeds that can lead to high genetic diversity and heterozygosity [69,70].

### Geographic differentiation/IBD?/Connectivity

With regard to our results, we found strong genetic differentiation between Iranian sites of *Potamogeton pectinatus*. Mountains act as strong genetic barriers but river valleys recognized as corridors for gene flow [10]. The distribution of Iranian *Potamogeton* seems to be linked to mountain regions as indicated by the STRUCTURE results (for 10 populations) (Fig 6) and by the PCoA at individual level (for all 36 sites) (Fig 3). We obtained clear groups according to their ‘Mountain positions’: NE Alborz (3); NW Alborz (2); alongside of Urmieh lake (21, 24); SW Zagros (19, 27, 30), SE Zagros (22), N Zagros (34) and Kerman (35). Also allelic differentiation (*D*_est_ values) showed very low or absent connectivity across mountains north (Albroz) and south (Zagros/Kerman).

A strong correlation between genetic and geographic distances revealed a pattern of IBD across the distribution range of *P*. *pectinatus* in Iran. We found a strong positive correlation between *D*_est_ and geographic distances (Fig 5). The differentiation was larger for populations separated by the Alborz mountains than those separated by the Zagros mountains.

In spite of short (direct flight) distances between some populations, a high level of differentiation occured because of dispersal barriers such as Alborz mountain (Amirkelaieh (2), Azbaran(3)) or (Kaniborazan (21); Miandoab (24)). The highest differentiation was between Azbaran and Yaschaman (*F*_ST_ = 0.595; *D*_est_ = 0.925) (Table 4). The combination of Alborz mountain, Kerman mountains and Kavir desert most likely explain this value.

There is however some connectivity between both sides of Zagros mountains namely between SW Zagros (19, 25, 27, 30) and N Zagros (4, 5, 6, 7, 8, 9, 18 etc.). This area in Iran was reported as a route for bird migration [71]. It thus can be assumed that e.g. between Izeh and Isfahan this route forms a ridge between high Zagros and low Zagros. Equally important could be the wider pass with upstream rivers reaching geographically rather close towards the watershed. A connectivity over longer distance exists between Vimcheh in Isfahan (N. Zagros) and Yaschaman (Kerman) with the corridor on the same side of the mountain area as a probable explanation (= ITS-B and cpDNA-380), together with the bird migration maintaining this connectivity. The connectivity along the same side of Zagros Mts is higher than for all other pairwise comparisons. The populations South of Zagros Mts, showed low differentiation between SW Zagros and SE Zagros and this was confirmed by the STRUCTURE results. Recent studies also indicate a plant species migration along altitudinal gradients [11]. A study of the population genetic diversity of *Rhodiola dumulosa* (Crassulaceae) between rivers in Mountains ridges also showed strong differentiation [5]. From that PCoA of individuals, there is a clear geographical gradient along the first axis and corresponding to their position alongside or across mountains.

### Microsatellites with dispersal evidence from maternal cpDNA of trnH-psbA

The most common haplotype (cp 378) can be observed in most regions but cp 379 in only two regions (NW Alborz;up of Lake Urmieh and N.Zagros) despite their large allelic differentiation of microsats. Thus the mutation of the chloroplast mononucleotide repeat (1 base only) is most likely independent as event in both regions and not an indication of long distance dispersal. Another shorter variant (cp377) also occurs in only two regions (NE of Alborz and N.Zagros but remain very different for their microsats. So again, it most likely is an independent mutation event in both regions and not necessarily an indication of long distance dispersal. However, the haplotype variant cp380 and combined with a unique nuclear ITS-B variant could be an indication of historical dispersal over longer distances. These slow evolving genes are similar but the faster evolving microsats show large differences between pop 1, 5 and 15/35. Therefore, we assume that the isolation of 15/35 is not a recent one. *Potamogeton* represents the largest genus in Potamogetonaceae, including about 100 species and 50 interspecific hybrids worldwide. Hybridization therefore can be another complexity that is thought to be relatively frequent, because complex series of polyploidy and aneuploidy are present within the genus [28]. The hexaploid *P*. *pectinatus* having 78 chromosomes could form hybrids with other linear leafed taxa such as *P*. *filiformis* and *P*. *amblyphyllus* [72]. This issue might have an effect on the genetic diversity of *Potamogeton* populations. In our study we have detected only *P*. *pectinatus* haplotypes in correlation with specific microsatellite alleles for each site. No chloroplast capture was found. Consequently, we assume the absence of hybrid specimen in our samples.

### Habitat

It is proven that habitat diversity often makes ecological barriers against gene flow that leads to interpopulation genetic separation [73,74]. Different populations produce different selection pressures in the presence of environmental conditions [75,73]. Iran has different ecological conditions that lead to different plant vegetations and biogeographic regions. In recent years, changing environmental conditions such as global warming, drought or in some cases grazing by fishes could be interesting for knowing how genetic diversity is retained in such situations [74]. Iran lies in a region that confronts droughts. This phenomenon could have led to the bottleneck effect in Vimcheh and Izeh because of temporary habitats at low level of river water. In Azbaran, there is a high level of fish grazing pressure and also probably water bird feed on seeds and tubers that might have led to repeated bottlenecks (Table 3). The highest allelic and heterozygosity level was observed in N.Alborz and Kaniborazan population in West of Alborz-Azarbayejan Province. These provinces is documented to harbor the largest ecological diversity of Iran. Assignment of individuals to the own Kaniborazan population was highest because this population had the highest number of private alleles. In aquatic ecosystems, flow regimes and seasonal changing of water level lead to spread the seed to different habitats, inducing genetic diversity of aquatic populations [40]. Our results showed few shared alleles giving a stronger population genetic structure. The flora of the mountains provides many examples of closely related species or subspecies with populations that show limited gene flow due to these inherent geographic barriers [7,8]. In this study the individuals corresponding to populations of Aligoodarz, Borujen, Yaschaman and Googhar were separated from other *P*. *pectinatus* populations with 100% bootstrap. Additional evidence from ITS and cpDNA was provided and indicated these as an evolutionary significant unit (ESU). The morphological characters such as leaf sheath, width of leaf and tip of leaf were different from all other populations. These populations clearly belong to an evolutionary lineage within *P*. *pectinatus* and do not represent another species (e.g. *P*. *amblyphyllus*) because their ITS in a NJtree was separated (97% bootstrap) from *P*. *amblyphyllus* (*S*. *amblyophylla*) of NCBI (next paper under submission).

As a conclusion, when considering *P*. *pectinatus* populations over a long gradient of regions across mountain ranges and around deserts in Iran, their genetic differentiation over distances was more pronounced and structured than for temperate regions. However, across such habitats *P*. *pectinatus* maintained an even higher level of allele and gene diversity than in temperate regions.
